# Assessment of Pediatric Residents in the United States by Sex, Ethnoracial and Intersectional Group

**DOI:** 10.5334/pme.2862

**Published:** 2026-07-21

**Authors:** Hannah L. Kakara Anderson, Daniel C. West, Alan J. Schwartz, Laura Lockwood, Caroline E. Rassbach, Ariel S. Winn, Catherine D. Michelson, Benjamin Kinnear, Jerome G. Chen, Abigail Martini, David A. Turner, Daniel J. Schumacher

**Affiliations:** 1 The Children’s Hospital of Philadelphia (CHOP), Philadelphia, PA, US; 2 Perelman School of Medicine at the University of Pennsylvania, Philadelphia, PA, US; 3 Maastricht University School of Health Professions Education, Maastricht, NL; 4 CHOP Education Collaboratory, Philadelphia, PA, US; 5 University of Illinois Chicago, Chicago, IL, US; 6 Association of Pediatric Program Directors Longitudinal Educational Assessment Research Network, McLean, VA, US; 7 University Colorado School of Medicine, Denver, CO, US; 8 Stanford University School of Medicine, Palo Alto, CA, US; 9 Harvard Medical School, Boston, MA, US; 10 Northwestern Feinberg School of Medicine, Chicago, IL, US; 11 Cincinnati Children’s Hospital Medical Center, Cincinnati, OH, US; 12 University of Cincinnati College of Medicine, Cincinnati, OH, US; 13 Orlando Health Arnold Palmer Hospital for Children, Orlando, FL, US; 14 Alabama College of Osteopathic Medicine, Dothan, AL, US; 15 American Board of Pediatrics, Chapel Hill, NC, US

## Abstract

**Introduction:**

Inequitable assessments in medical education disproportionately affect historically marginalized trainees and undermine development of a diverse physician workforce. With programs around the world incorporating competency-based medical education (CBME), understanding whether assessment disparities exist in specialty-specific competency frameworks is critical. This study examined disparities in program-reported performance assessments of pediatric residents across demographic groups in two pediatric-specific competency frameworks: the General Pediatrics Entrustable Professional Activities (EPAs) and Accreditation Council for Graduate Medical Education (ACGME) pediatric milestones.

**Methods:**

This prospective cohort study was conducted at 15 pediatric residency programs in the U.S. across the 2021–22, 2022–23, and 2023–24 academic years. Clinical Competency Committee-assigned entrustment-supervision levels for 5 EPAs and all milestone levels were collected twice yearly. Residents self-reported ethnoracial group and sex, analyzed as exposure to systems of racism and sexism. Time-to-event analysis and growth curve modeling with intersectional analytic approaches examined disparities across demographic groups.

**Results:**

475 of 803 (59%) residents completed demographic surveys, including 17.3% underrepresented in medicine (URiM), 22.9% Asian, 56.4% white, 70.7% female, and 27.8% male. Time-to-event analyses revealed significant differences for Asian and Asian female residents in reaching the highest EPA supervision level across all EPAs. Growth curve analyses showed Asian males had significantly flatter growth trajectories for EPA 15 (Lead an Interprofessional Team), resulting in lower estimated scores at graduation. URiM female residents had significantly lower scores on EPA 16 (Facilitate Handovers) at graduation. URiM residents showed lower Milestone growth curves for interpersonal communication, and Asian residents for systems-based practice.

**Discussion:**

Subtle but meaningful disparities exist in program-reported assessments of pediatric residents, with distinct patterns affecting Asian and URiM residents across both frameworks. These findings highlight the need for proactive attention to equity in competency-based medical education assessment systems.

## Introduction

The foundation of competency-based medical education (CBME) centers on ensuring that graduates of a training program are prepared to perform the activities of their profession, thereby meeting the needs of the patients they will serve [1]. A critical component of any CBME program is the assessment of trainees to ensure these outcomes are being met [2,3]. Based on these assessments, CBME argues that each trainee may need more or less time in training to meet desired outcomes, as opposed to rigid, time-based systems of education that assume outcomes will be met by the end of a specified training period [1,3]. Clinical Competency Committees (CCCs) routinely assess trainee progress using the Milestones from the Accreditation Council for Graduate Medical Education(ACGME), and the United States pediatrics community has increasingly adopted Entrustable Professional Activities (EPAs) to define patient-centered training outcomes as well [4,5].

Despite the promise of CBME to center the medical education system on patient-centered outcomes and allow for time-variable training, significant barriers impede its full operationalization – most notably, that assessment is often inequitable [3]. Inequity in assessment is a type of harmful discrimination which includes differences in quantitative ratings and qualitative comments (verbal and written) that do not reflect differences in performance but rather reflect disparities in access to learning and assessment, inappropriate or inadequate treatment, and/or bias [6]. There is no single attributable cause for these differences; mechanisms that produce inequitable assessment outcomes are highly varied and complex and may include unjust policies and procedures, implicit bias among assessors/raters, inaccessible learning environments which unequally disadvantage learners who are women and those from marginalized backgrounds, and many more [7,8,9,10,11,12,13,14]. Inequitable assessment outcomes have been quantitatively measured and qualitatively described in learners’ narrative evaluations, ratings, and advancement through training in both Undergraduate (UME) and GME [7,8,9,10]. These inequities tend to accumulate over the course of training and have long-lasting harmful impacts on learner advancement and career trajectory (e.g., limiting career options, impacting psychological wellness, etc.) [7,11]. Importantly, these harmful impacts on learners have downstream harmful impacts on society by hampering the development of a diverse physician workforce, which in turn leads to inequities in care of patients [12,13].

As pediatric programs continue to advance CBME and now begin to adopt the EPAs, proactive attention to equity is essential [14]. Pediatrics as a specialty is not immune to inequities seen across health professions education; for example, residents who are underrepresented in medicine (URiM), Asian, or female report experiencing significant inequity in verbal and written assessments [15]. Additionally, there is evidence of disparities in program-reported assessments for pediatric residents on the ACGME Pediatric Milestones [16]. However it is unknown if there are similar disparities in program-reported EPAs. If present, these disparities could risk compounding inequity and worsening long-term impacts on historically marginalized trainees. This study examined disparities in program-reported performance assessments of pediatric residents across demographic groups in two pediatric-specific competency frameworks: the General Pediatrics Entrustable Professional Activities (EPAs) and Accreditation Council for Graduate Medical Education (ACGME) pediatric milestones. This is a critical step to ensuring pediatric GME programs are addressing harmful discrimination and fulfilling their role of preparing a pediatric workforce best prepared to care for society.

## Methods

### Setting and Data Sources

We conducted a prospective cohort study. During the 2021–22, 2022–23, and 2023–24 academic years, as part of a larger study on CBME, we collected CCC-assigned entrustment-supervision levels for the 17 General Pediatrics Entrustable Professional Activities (EPAs) [4] as well as milestone levels for the 22 Accreditation Council for Graduate Medical Education (ACGME) pediatric milestones [17] for pediatric residents at 15 pediatric residency programs twice per year. EPAs were recorded on a 5-level supervision scale: Level 1 (“Trusted to observe only”), Level 2 (“Trusted to execute with direct supervision and coaching”), Level 3 (“Trusted to execute with indirect supervision and discussion of information”), Level 4 (“Trusted to execute with indirect supervision and may require discussion of information but only for selected complex cases”), Level 5 (“Trusted to execute without supervision”). These are reported on a 1–8 scale, corresponding to Levels 1, 2a, 2b, 3a, 3b, 4, 5a, and 5b. We focused on the EPA supervision scale Levels 3, 4, and 5 because these represent significant changes in supervision that typically occur over the course of GME training (Box 1). For the EPAs, we focused on the five EPAs (EPA 4, 5, 10, 15, 16) because these are the most commonly assessed by programs.

**Box 1 Five General Pediatric EPAs Most Commonly Assessed by Programs.**

EPA 4: Manage Patients with Acute, Common Diagnoses in an Ambulatory, Emergency, or Inpatient Setting

EPA 5: Provide a Medical Home for Well Children of All Ages

EPA 10: Resuscitate, Initiate Stabilization of the Patient, and Then Triage to Align Care with Severity of Illness

EPA 15: Lead an Interprofessional Health Care Team

EPA 16: Facilitate Handovers to Another Health Care Provider Either Within or Across Settings

For the Milestones, we included all program-reported competency domains, including interpersonal communication skills (ICS), medical knowledge (MK), practice-based learning and improvement (PBLI), patient care (PC), professionalism (PROF), and systems-based practice (SBP). Domain levels are reported on a 1–9 scale, corresponding to milestone levels 1, 1.5, 2, 2.5, 3, 3.5, 4, 4.5, and 5. Level descriptions vary depending on the competency domain, but represent movement from novice to expert in the specialty. We focused on Levels 3, 4, and 5 because these represent significant changes in competency over the course of residency.

### Predictor Variables and Demographics

From May 2023-June 2024, we surveyed residents in the 15 programs asking them to self-report their demographic characteristics. Demographic characteristics were grouped in the following self-identified groups: ethnoracial group (white, URiM [African American/Black, Hispanic, Native American/Alaskan Native, Native Hawaiian/Pacific Islander], Asian), and sex. The URiM group was created based on prior literature [9]. Respondents to more than one racial category were included in all selected. Respondents who reported transgender or other (fill in the blank) responses for sex were too small in number to analyze and were excluded. Data were only included if residents completed the entire survey. This study followed the Strengthening the Reporting of Observational Studies in Epidemiology (STROBE) reporting guidelines for cohort studies [18]. The study was reviewed and deemed exempt by the Cincinnati Children’s Institutional Review Board.

### Statistical Analyses

We employed two different approaches to analyze the relationship between EPAs, Milestones, and demographic groups. First, we used a time to event analysis to understand any differences in time to reach assessed higher levels of competence in the EPAs and Milestones among residents of different demographic categories. Time to event analysis is particularly useful for understanding time to reach levels of assessed supervision in the EPAs and are a generally accepted form of analyzing growth in individual residents’ competence over time. Second, we used growth curve analysis (hierarchical linear mixed effects) to understand any differences in growth trajectories in the EPAs and Milestones over time. Hierarchical linear mixed effects have been used to examine disparities in Milestones in other specialties; this analysis allows us to situate pediatrics within findings from other specialties in the literature [19].

In creating models, we adopted an intersectional analytic approach, meaning that: 1) we considered demographics as potentially multiple and overlapping; 2) we used historically marginalized groups (URiM, female) as the reference categories for analysis, where possible, rather than assuming majority groups as default reference categories; and 3) we considered self-reported demographic characteristics of race/ethnicity and sex as proxy for exposure to systems of racism and sexism (random effects rather than essentialist traits) [20,21]. Due to the smaller numbers in intersectional groups (i.e., groups of both race/ethnicity and sex such as Asian Female), we present intersectional findings as exploratory.

All tests were two-tailed. All analyses comprise 17 separate regression models, each addressing a distinct outcome or subgroup. Within each model, coefficient-level p-values reflect the presence of other predictors and were not subjected to further cross-model correction. The significance level was set at 0.05 for all tests.

#### Time to event analysis

To understand any differences in time to reach assessed higher competence among demographic groups, we first modeled the relationship between demographic groups (plus two intersectional groups [URiM and female, Asian and female]) and time to reach levels 3, 4, and 5 on the general pediatrics EPAs. Second, we modeled the relationship between demographic groups and time to reach levels 3, 4, and 5 on the Pediatrics Milestones. Aligned with our intersectional approach, we used historically marginalized groups (URiM, female) as the reference categories for analysis. We included a random program intercept to adjust for residents clustered in programs.

#### Growth curve analysis

We employed hierarchical linear mixed-effects models to estimate any differences in growth trajectories in EPA and Milestone scores among demographic groups over time. Time was modeled as a continuous variable representing months since the start of residency; we used a quadratic function of time. Separate models were constructed for both EPAs and Milestones. In Model 1, the primary outcome was the EPA score at Fall and Spring (corresponding to twice-yearly CCC reporting). Fixed effects included time, sex, race/ethnicity, and their interactions with time to assess disparities in both baseline assessments and rates of growth. For Milestones, to be able to compare slopes with existing literature, we used historically dominant groups (white, male) as reference categories; this is accepted within intersectional approaches for ease of interpretation and to compare with historical findings [22]. Random effects to adjust for residents (intercepts and slopes) clustered in programs were included in all models. We used likelihood ratio tests and information criteria to compare model fit.

## Results

Overall, 475 of 803 (59%) pediatric residents completed the demographic survey and were included in analyses (URiM [17.3%]; Asian [22.9%]; white [56.4%]; female [70.7%]; male [27.8%]) (Table 1).

**Table 1:** Demographic Characteristics of Pediatric Residents (N = 475).


CHARACTERISTIC	N (%)

Ethnoracial Group^1^:	

Asian	109 (22.9%)

URiM^2^	82 (17.3%)

White	268 (56.4%)

Other/Decline	16 (3.4%)

Sex	

Female	336 (70.7%)

Male	132 (27.8%)

Trans/Other	7 (1.5%) [too small to analyze]

Intersectional Group	

URiM Female	59 (13.0%)

Asian Female	81 (17.8%)

White Female	187 (41.2%)


URiM = underrepresented in medicine. ^1^ Residents could self-identify as more than one. ^2^ Includes African American or Black, Hispanic, Native American or Alaskan Native, and Native Hawaiian or other Pacific Islander.

Time-to-event analyses of EPA’s showed some significant differences in reaching supervision scale level 5 in averages of all EPAs for Asian residents and Asian female residents; no significant differences were found in reaching supervision scale level 3 or 4 for any demographic groups in averages of all EPAs (Table 2a). Time-to-event analyses of Milestones showed no significant differences in reaching Milestone Levels 3, 4, and 5 for any demographic groups in all Milestones (Table 2b).

**Table 2a:** Time to Event Analysis of Supervision Levels 3, 4, 5 for EPAs (4, 5, 10, 15, and 16) by Demographic Group.


DEMOGRAPHIC GROUP	SUPERVISION SCALE LEVEL 3			SUPERVISION SCALE LEVEL 4			SUPERVISION SCALE LEVEL 5		
		
	ESTIMATE	MODEL COEFFICIENT [95% CI]	p-VALUE	ESTIMATE	MODEL COEFFICIENT [95% CI]	p-VALUE	ESTIMATE	MODEL COEFFICIENT [95% CI]	p-VALUE

Ethnoracial Group^1^									

URiM^2^ [reference]	–	–	–	–	–	–		–	–

Asian	–0.00	–0.35 – 0.34	0.10	–.014	–0.50 – 0.22	0.45	–0.72	–1.21 – –0.22	**0.01**

White	–0.00	–0.29 – 0.28	0.98	0.00	–0.28 – 0.28	0.99	–0.23	–0.62 – 0.15	0.23

Sex:									

Female [reference]	–	–	–	–	–	–		–	–

Male	0.23	–0.07 – 0.52	0.16	0.00	–0.30 – 0.31	0.98	–0.25	–0.66 – 0.16	0.24

Intersectional Group									

URiM Female [reference]	–	–	–	–	–	–	–	–	–

Asian Female	–0.26	–0.66 – 0.13	0.19	0.14	–0.27 – 0.55	0.51	0.68	0.12 – 1.25	**0.02**

White Female	–0.20	–0.54 – 0.13	0.23	–0.05	–0.39 – 0.28	0.76	0.18	–0.28 – 0.64	0.44


URiM = underrepresented in medicine; CI = confidence interval. ^1^ Residents could self-identify as more than one. ^2^ Includes African American or Black, Hispanic, Native American or Alaskan Native, and Native Hawaiian or other Pacific Islander.

**Table 2b:** Time to Event Analysis: Milestones Levels 3, 4, 5 for All Milestones (Interpersonal Communication Skills (ICS), Medical Knowledge (MK), Practice-Based Learning and Improvement (PBLI), Patient Care (PC), Professionalism (PROF), System-based Practice (SBP) by Demographic Group.


DEMOGRAPHIC GROUP	MILESTONE LEVEL 3			MILESTONE LEVEL 4			MILESTONE LEVEL 5		
		
	ESTIMATE	MODEL COEFFICIENT [95% CI]	p-VALUE	ESTIMATE	MODEL COEFFICIENT [95% CI]	p-VALUE	ESTIMATE	MODEL COEFFICIENT [95% CI]	p-VALUE

Ethnoracial Group:^1^									

URiM^2^ [reference]	–	–	–	–	–	–	–	–	–

Asian	0.11	–0.45 – 0.67	0.71	0.10	–0.75 – 0.95	0.82	–1.52	–3.73 – 0.68	0.18

White	0.36	–0.10 – 0.81	0.12	0.50	–0.18 – 1.17	0.15	0.92	–0.45 – 2.28	0.19

Sex:									

Female [reference]	–	–	–	–	–	–	–	–	–

Male	0.29	–0.18 – 0.76	0.22	0.30	–0.41 – 1.00	0.41	0.53	–0.98 – 2.03	0.49

Intersectional Group:									

URiM Female [reference]	–	–	–	–	–	–	–	–	–

Asian Female	–0.32	–0.96 – 0.32	0.32	–0.05	–1.01 – 0.91	0.92	1.59	–0.89 – 4.07	0.21

White Female	–0.41	–0.93 – 0.11	0.13	–0.40	–1.18 – 0.37	0.31	–0.60	–2.24 – 1.04	0.47


URiM = underrepresented in medicine; CI = confidence interval. ^1^ Residents could self-identify as more than one. ^2^ Includes African American or Black, Hispanic, Native American or Alaskan Native, and Native Hawaiian or other Pacific Islander.

Growth curve analyses of the EPA’s showed the growth curves for EPA 15 (Lead an Interprofessional Team) were significantly flatter for Asian males compared to white males (the reference group); although Asian males had significantly higher supervision level scores at the beginning than other groups, these flatter growth trajectories resulted in lower estimated marginal scores at graduation than other groups on this EPA (Table 3a). URiM female residents had significantly lower supervision level scores on EPA 16 (Facilitate Handovers to Another Health Care Provider) than other groups at graduation, but their overall curves did not otherwise differ.

**Table 3a:** Growth Curve Analysis: Supervision Levels 3, 4, 5 for EPA 4, 5, 10, 15, 16 by Demographic Group.


PGY	GROUP	EPA 4 ESTIMATE [CI]	*P*	EPA 5 ESTIMATE [CI]	*P*	EPA 10 ESTIMATE [CI]	*P*	EPA 15 ESTIMATE [CI]	*P*	EPA 16 ESTIMATE [CI]	*P*

Fall	Asian Female	5.18 [4.66, 5.70]	*0.36*	4.26 [3.24, 5.29]	*0.18*	3.23 [2.32, 4.13]	*0.55*	2.03 [1.34, 2.72]	*0.23*	4.17 [3.26, 5.09]	*0.245*

	Asian Male	5.44 [4.54, 6.34]	*0.27*	3.30 [1.74, 4.86]	*0.66*	**4.44 [3.04, 5.84]**	***0.035***	**3.24 [2.06, 4.42]**	***0.007***	4.72 [3.42, 6.03]	*0.10*

	URiM Female	4.85 [4.38, 5.32]	*0.83*	3.74 [2.77, 4.71]	*0.81*	3.35 [2.49, 4.20]	*0.35*	1.95 [1.31, 2.59]	*0.29*	4.27 [3.39, 5.14]	*0.13*

	URiM Male	4.81 [3.96, 5.65]	*0.81*	4.39 [2.92, 5.87]	*0.30*	3.37 [2.05, 4.69]	*0.55*	2.14 [1.05, 3.24]	*0.33*	4.04 [2.81, 5.28]	*0.60*

	White Female	4.83 [4.46, 5.20]	*0.71*	3.66 [2.81, 4.52]	*0.93*	3.11 [2.37, 3.85]	*0.68*	1.88 [1.36, 2.40]	*0.29*	4.04 [3.24, 4.84]	*0.30*

	White Male	4.91 [4.44, 5.39]		3.64 [2.66, 4.61]		2.98 [2.12, 3.83]		1.58 [0.94, 2.23]		3.75 [2.86, 4.63]	

Spring	Asian Female	7.79 [7.23, 8.35]	*0.32*	6.69 [5.70, 7.67]	*0.21*	6.97 [6.12, 7.83]	*0.82*	8.15 [7.48, 8.82]	*0.43*	7.89 [6.99, 8.79]	*0.65*

	Asian Male	7.83 [6.92, 8.74]	*0.44*	5.16 [3.79, 6.53]	*0.18*	6.69 [5.51, 7.86]	*0.52*	**7.05 [6.02, 8.09]**	***0.013***	7.46 [6.28, 8.63]	*0.64*

	URiM Female	7.11 [6.39, 7.82]	*0.44*	5.89 [4.74, 7.04]	*0.75*	6.45 [5.46, 7.45]	*0.21*	8.80 [7.96, 9.64]	*0.47*	**6.53 [5.52, 7.55]**	***0.010***

	URiM Male	7.28 [6.25, 8.32]	*0.80*	6.59 [5.09, 8.10]	*0.49*	7.22 [5.93, 8.50]	*0.82*	8.65 [7.51, 9.80]	*0.76*	7.38 [6.11, 8.66]	*0.58*

	White Female	7.84 [7.40, 8.29]	*0.20*	6.24 [5.36, 7.13]	*0.69*	6.76 [6.00, 7.52]	*0.40*	8.54 [7.98, 9.10]	*0.83*	7.40 [6.57, 8.22]	*0.35*

	White Male	7.43 [6.85, 8.02]		6.08 [5.06, 7.09]		7.07 [6.19, 7.94]		8.46 [7.76, 9.17]		7.71 [6.80, 8.63]	


URiM = underrepresented in medicine (Includes African American or Black, Hispanic, Native American or Alaskan Native, and Native Hawaiian or other Pacific Islander); CI = confidence interval.

Growth curve analysis of the Milestones showed there were no effects of demographics on the shapes of the growth curves for any Milestones (Table 3b). However, growth curves of URiM residents were lower overall for Interpersonal Communication Skills (ICS) (B = –0.43, 95% CI [–0.85,–0.02], p = 0.04), and growth curves of Asian residents were lower overall for Systems Based Practice (SBP) (B = –0.32, 95% CI [–0.56,–0.08], p = 0.008) than growth curves of white residents.

**Table 3b:** Growth Curve Analysis of Milestones Levels for all Competencies (Interpersonal Communication Skills (ICS), Medical Knowledge (MK), Practice-Based Learning and Improvement (PBLI), Patient Care (PC), Professionalism (PROF), System-based Practice (SBP) by Demographic Group.


GROUP	ICSESTIMATE [CI]	*P*	MKESTIMATE [CI]	*P*	PBLIESTIMATE [CI]	*P*	PCESTIMATE [CI]	*P*	PROFESTIMATE [CI]	*P*	SBPESTIMATE [CI]	*P*	AVGESTIMATE [CI]	*P*

**Ethnoracial Group:¹**														

White [reference]	–	*–*	–	*–*	–	*–*	–	*–*	–	*–*	–	*–*	–	*–*

URiM²	–0.43 [–0.85,–0.02]	*0.04*	3.06 [(–5.44 – 11.56)]	*0.48*	–1.57 [(–13.02 – 9.87)]	*0.79*	–3.89 [(–11.95 – 4.17)]	*0.34*	0.00 [(–9.47 – 9.48)]	*0.99*	0.78 [(–5.76 – 7.32)]	*0.82*	–0.36 [(–7.04 – 6.32)]	*0.92*

Asian	–2.13 [(–12.69 – 8.42)]	*0.69*	–5.31 [(–14.02 – 3.41)]	*0.23*	–7.56 [(–19.11 – 3.99)]	*0.20*	–5.31 [(–13.67 – 3.05)]	*0.21*	0.41 [(–9.25 – 10.07)]	*0.93*	–0.32 [–0.56, –0.08]	0.008	–3.64 [(–10.54 – 3.26)]	*0.30*

**Sex**														

Male [reference]	–	*–*	–	*–*	–	*–*	–	*–*	–	*–*	–	*–*	–	*–*

Female	2.58 [(–2.67 – 7.82)]	*0.34*	0.82 [(–3.62 – 5.26)]	*0.72*	–2.26 [(–8.25 – 3.73)]	*0.46*	0.32 [(–3.81 – 4.46)]	*0.88*	2.07 [(–2.86 – 6.99)]	*0.41*	–0.14 [(–3.53 – 3.25)]	*0.94*	0.77 [(–2.65 – 4.18)]	*0.66*

**Intersectional Group:**														

URiM Female	–6.06 [(–17.82 – 5.70)]	*0.31*	–6.39 [(–16.32 – 3.54)]	*0.21*	3.45 [(–9.93 – 16.83)]	*0.61*	1.98 [(–7.36 – 11.33)]	*0.68*	–1.56 [(–12.62 – 9.50)]	*0.78*	–0.86 [(–8.47 – 6.75)]	*0.82*	–1.21 [(–8.97 – 6.55)]	*0.76*

Asian Female	–2.49 [(–14.47 – 9.49)]	*0.68*	3.94 [(–6.00 – 13.87)]	*0.44*	5.94 [(–7.26 – 19.15)]	*0.38*	2.13 [(–7.37 – 11.62)]	*0.66*	–2.96 [(–13.98 – 8.05)]	*0.60*	2.60 [(–5.10 – 10.30)]	*0.51*	1.21 [(–6.63 – 9.04)]	*0.76*


URiM = underrepresented in medicine (Includes African American or Black, Hispanic, Native American or Alaskan Native, and Native Hawaiian or other Pacific Islander); CI = confidence interval.

## Discussion

Our prospective, multi-year study of a nationally geographically representative sample found that there are subtle, small but unique disparities in CCC-level program-reported performance assessments of pediatric residents. Specifically, in our time-to-event analyses of program-reported assessment milestones, there were no differences in time to reach any of the Pediatric Milestones levels based on group but some differences in time to reach the highest EPA levels specifically for Asian residents. In our growth curve analyses of program-reported performance assessments, we found small but significant differences in the growth curves of both the general pediatrics EPAs and Pediatric Milestones including specifically differences in growth curves in mean scores for URiM and Asian residents in ICS and SBP, respectively. While we present intersectional group analyses, we caution that these findings are exploratory in nature due to the small sizes of these groups.

Situating our findings in existing evidence, population-level differences found in this study do not reflect actual differences in performance but rather reflect longstanding inequities in assessment and should not be interpreted as deficits in individual residents or these groups. Similar disparities in URiM residents’ program-reported performance assessments of older Milestones used in pediatrics, emergency medicine, and internal medicine specialties draw attention to how the Milestones may have similar disparities in scores even across specialties [12,16]. In contrast to other specialties, it is unclear why Asian residents experienced disparities in our study compared with residents from other groups. It is possible that program-level and national efforts to combat inequity may need increased awareness that Asian residents are also subject to inequity. As programs increasingly implement and rely on competency frameworks, future research should synthesize and investigate these findings. Further, in response to these findings, programs must consider how to respond to and potentially mitigate these disparities: in an ideal CBME system, time-variable training would allow for more time for trainees who need more support or time to reach desired outcomes. This flexibility may allow for the amelioration of any differences in scores by the conclusion of training but could have unrelated unintended effects.

Previous research within pediatrics also shows that pediatric residents report experiencing significant inequity in frontline assessments [15]. The disparities reported in this study of CCC assessments are relatively subtle and do not align exactly with self-reported data from pediatric residents; however, this paradox could be illuminated, in part, by two considerations from the literature. First, this study only examined CCC-level decisions collected twice-yearly and may not reflect what trainees experience in frontline workplace-based assessment on any given day. A CCC may choose to factor in or disregard individual workplace-based assessments that are completed by physician assessors; CCCs do adjust scores based on group consensus and may adjust for perceived biases or inequalities in individual workplace-based assessment data [23,24]. It is also known that CCCs routinely consider personal knowledge of residents and previously undocumented data when making summative assessment decisions [25]. Finally, it is also known that intentionally designed CCCs’ do seek to adopt best practices to mitigate bias, such as developing diverse committee membership and proactive attention to equity [26,27]. While trainees may or may not be aware of the CCCs’ activities and may not feel the effects of equity or inequity at the CCC level, it is possible that trainees experience inequity in individual frontline assessments while the CCC is able to compensate or adjust for these. This highlights the potentially critical role of the CCC in ameliorating inequities in performance assessment [28,29]. Future studies should look at disparities in frontline assessments, how these may or may not correlated with CCC-level ratings, and CCC-level practices that may protect against or exacerbate inequity.

Second, situating these quantitative findings within emerging qualitative research shows that identifying the presence of inequity may not be as simple as measuring disparities or differences [6]. Traditional quantitative measures of inequity (like comparing test scores, grades, or assessment ratings between demographic groups) may miss inequities that residents experience. Some residents who face inequitable treatment may still receive positive scores and comments on paper. This means that if researchers only look at numerical outcomes, they might conclude no inequity exists when inequity is indeed present but hidden [30].

This study has limitations. First, it relies on self-reported demographic data which could be incomplete and is subject to social response bias; we do not have information on non-responders. However, this self-reported data may be more current than program-reported demographic data, and the distribution of respondents does align with overall workforce demographics for pediatrics [31]. Second, the American Board of Pediatrics has recently released a new, shorter set of EPAs for General Pediatrics in summer of 2025; this study reports on the previous version of the EPAs, which were current at the time of the study design and execution. Third, several race/ethnicity categories were lumped into a single URiM group for analysis: this was done to improve power in analysis and does align with existing literature as an appropriate practice for measuring assessment disparities [12]; however, it does limit specificity in our findings. Fourth, we could not perform an a priori sample size calculation. However, based on the observed variation and sample size, we have 80% post hoc power to detect a hazard ratio (e.g. for time-to-reach-EPA-level-4) of 1.66 between male and female residents and 1.8 between URIM and non-URIM residents. Finally, it is known that programs have variability in adherence to CBME in implementation, and many of the programs participating in this study may have newly been using EPAs. Implementation of CBME in the U.S. is still far from complete, and even if programs share the common competency frameworks for assessment of the EPAs and Milestones, they may or may not follow the other five critical components of CBME, including workplace-based assessment, programmatic assessment, etc., all of which have potential to influence the findings of this study.

## Conclusion

This multi-year study reveals that subtle but unique disparities exist in program-reported performance assessments of pediatric residents, with particular patterns affecting Asian and URiM residents in specific EPA and Milestone assessments. While these disparities are relatively small and may not align directly with residents’ self-reported experiences of inequity, they highlight the complex interplay between frontline assessment and Clinical Competency Committee assessment determinations. The findings suggest that CCCs may play a crucial role in mitigating some inequities through their review processes, yet disparities persist in ways that warrant attention. This aligns with current calls for attention to the importance of robust implementation of CBME that incorporates both rigorous, equitable frontline workplace-based assessment and rigorous CCC processes. As pediatric programs increasingly implement CBME, these results underscore the need for continued vigilance in identifying and addressing assessment disparities that may be subtle yet significant. Future research should integrate quantitative disparity measures with qualitative investigations of lived experiences, explore differences and relationships between frontline workplace-based assessments and CCC determinations, and synthesize findings across specialties to develop a more comprehensive understanding of equity in medical education assessment.
